# Standardization of Plant Microbiome Studies: Which Proportion of the Microbiota is Really Harvested?

**DOI:** 10.3390/microorganisms8030342

**Published:** 2020-02-28

**Authors:** Abdoul Razack Sare, Gilles Stouvenakers, Mathilde Eck, Amber Lampens, Sofie Goormachtig, M. Haïssam Jijakli, Sebastien Massart

**Affiliations:** 1 Laboratory of Integrated and Urban Phytopathology, Gembloux Agro-Bio Tech, University of Liège, 5030 Gembloux, Belgium; G.Stouvenakers@uliege.be (G.S.); Mathilde.Eck@uliege.be (M.E.); mh.jijakli@uliege.be (M.H.J.); sebastien.massart@uliege.be (S.M.); 2 VIB-UGent Center of Plant Systems Biology, 9052 Ghent, Belgium; Amber.Lampens@psb.vib-ugent.be (A.L.); sogoo@psb.vib-ugent.be (S.G.); 3 Department of Plant Biotechnology and Bioinformatics, Ghent University, 9052 Ghent, Belgium

**Keywords:** plant microbiome, bias, harvesting protocol, standardization, apple fruit, lettuce roots

## Abstract

Studies in plant-microbiome currently use diverse protocols, making their comparison difficult and biased. Research in human microbiome have faced similar challenges, but the scientific community proposed various recommendations which could also be applied to phytobiome studies. Here, we addressed the isolation of plant microbiota through apple carposphere and lettuce root microbiome. We demonstrated that the fraction of the culturable epiphytic microbiota harvested by a single wash might only represent one-third of the residing microbiota harvested after four successive washes. In addition, we observed important variability between the efficiency of washing protocols (up to 1.6-fold difference for apple and 1.9 for lettuce). QIIME2 analysis of 16S rRNA gene, showed a significant difference of the alpha and beta diversity between protocols in both cases. The abundance of 76 taxa was significantly different between protocols used for apple. In both cases, differences between protocols disappeared when sequences of the four washes were pooled. Hence, pooling the four successive washes increased the alpha diversity for apple in comparison to a single wash. These results underline the interest of repeated washing to leverage abundance of microbial cells harvested from plant epiphytic microbiota whatever the washing protocols, thus minimizing bias.

## 1. Introduction

Plant tissues provide several niches for microbial growth and a rough distinction can be made between the aboveground plant organs, referred to as phyllosphere and the belowground microbial niches: the rhizosphere (the soil directly surrounding plant roots from which the physicochemical properties are influenced by the root); the rhizoplane (the root surface); and the root endosphere (the compartment formed by the apoplastic spaces between the root cells). Just as below-ground, the phyllosphere also comprises different compartments: the caulosphere, formed by the stems, the phylloplane, i.e., the leaf surface (with preferred habitats near nutrient rich specialized structures such as trichomes, stomata, and veins), the anthosphere, i.e., the compartment formed by the flowers, the carposphere, i.e., the habitat created by the fruits and the spermosphere, shaped by the seeds. 

Our current definitions of the different phyllosphere or root-associated communities are constrained by technical limitations (i.e., incomplete microbiome separation). The strength of the interaction of microbial cells with plants greatly varies within any single phytobiome. When considering the root system from the outside to the inside, the microbial diversity decreases while the degree of specialization and the strength of attachment and interaction increases [1]. Inhabitants of the rhizosphere exhibit several features, enabling them to colonize the root system [2]. It should be stressed that the diversity and the density of the bacterial community significantly varies between different regions along the root system [3]. Moreover, differentiation between rhizosphere and rhizoplane is unclear regarding the continuum of microbial population variation from outside to inside the roots and is subject to variability between studies. The phyllosphere also displays technical limitations. Phyllosphere is challenged by difficult conditions such as fluctuations in solar radiation, temperature, humidity, and heterogeneous availability of nutrients [4]. In order to survive the harsh environmental conditions and the oligotrophic environment, phyllosphere inhabitants, especially colonizers of the phylloplane, have developed specific features (e.g., pigments, motility, biofilm formation) and are populating specialized compartments such as substomatal chambers. The wax layer, the cuticle, and the spatial heterogeneity of the leaf play a pivotal role in shaping the phyllosphere microbial communities and their attachment to plant tissue [5]. Microorganisms residing on the fruit surface originate from the environment, or they gain access to this niche via other plant organs [6]. The production of biosurfactants and biofilm formation by the carposphere microbial communities to attach to the fruit surface has been demonstrated in earlier research [7]. The adherence to the fruit surface can vary significantly between microbes. Indeed, the strength of the interaction for some bacteria is influenced by the amount of wax in the fruit, environmental factors (temperature), discontinuities in the apple waxy cuticle (damaged tissue surrounding wounds) or stomatal opening. Moreover, plant microorganisms have special preference. They are not uniformly distributed on fruit surface, as it was shown with the fungal populations of the different parts of apple fruit [8].

Plant surfaces harbor very diverse and abundant bacterial and fungal communities that provide specific functions and traits. Consequently, these communities are considered as a key factor for plant growth and health [4,9]. In recent years, and thanks to high-throughput sequencing (HTS) Technologies, increasing attention has been paid to the understanding of the relationship between the plants and their microbial communities. 

Any microbiome study, also called phytobiome for plants, using HTS can include methodological biases at each step of the analysis, i.e., during: (i) microbiota harvesting, (ii) sample storage/preservation, (iii) sample preparation (DNA extraction and library preparation), (iv) sequencing, (v) bioinformatics analysis, and (vi) data repository and experiment documentation in databases [10,11,12]. In order to efficiently use the increasing resources devoted to phytobiome studies, it is therefore of prime importance to pay careful attention to these methodological biases [13,14,15,16]. Nevertheless, one bias of plant microbiome study has not been studied so far: the efficiency of the microbiota harvesting method and its effect on downstream molecular analyses. As shown in two papers on rhizosphere and rhizoplane microbiota sampling [13,17], there is currently a large diversity of protocol to harvest epiphytic microorganisms. Some authors suggested the use of standardized protocols, even before the advent of HTS [13,18]. International methods or standards of analysis (i.e., AOAC and ISO) exist but are related to specific microorganism studies such as human pathogens detection for food safety. Only a few papers compare protocols efficiency such as made by Richter-Heitmann et al. [19] on rice root microbiota and Donegan et al. [18] for *Enterobacter cloacae* recovering on bean leaves. A large diversity of protocols has been published so far to harvest epiphytic microbiota and Table 1 illustrates the diversity of protocols from two study cases: apple carposphere (12 protocols) and lettuce roots (4 protocols). Furthermore, a single washing step is commonly carried out without evaluating which fraction of the microbiota is really harvested and its representativeness of the whole community. The proportion of the microbiota which is harvested is barely ever mentioned whilst it is of utmost importance as it can generate quantitative or qualitative bias in the data interpretation during the downstream molecular analysis and can hamper comparison of results between studies. Therefore, in order to assess the impact of these parameters, we evaluated the effect of successive washes (with the same method and on the same plant sample) and of different washing protocols on the composition and quantity of microbiota harvested from apple carposphere and lettuce roots.

## 2. Materials and Methods

### 2.1. Microbiota Recovery

The microbiota of the apple fruit carposphere was harvested by four successive washes of whole apples of the commercial Golden Delicious cultivar (Marlene^®^) with four different washing methods found in the literature, and fully described in Table 2a. Briefly, it consisted of apple fruit shaking in (i) sterile distilled water (H_2_O) [24,37]; (ii) in kalium phosphate buffer and Tween (KPBT, pH 6.5) [28], a combination of shaking in (iii) KPBT (modified from [28]) or (iv) phosphate buffered saline (PBS) followed by 10 or 20 min of sonication respectively. For each method, four apples (stored at 4 °C) were washed together with one liter of buffer in a plastic bag, 4 times successively. The microbiota harvested by each successive wash was recovered and processed separately (4 methods * 4 successive washes = 16 microbiota sample per replicate). All the four methods were tested using apples originating from the same batch of commercial apple.

Lettuces seedling of 11 days old (var. Grosse Blonde Paresseuse, Semailles, Faulx-les-Tombes, Belgium) were grown in an aquaponic system (described by [38] coupling Nile tilapia (*Oreochromis niloticus* L.) farming and hydroponic crop cultivation. Root samples were taken one month later at the morphological stage of 34 leaves. Root samples were washed following the 4 different protocols described in Table 2b. Two protocols were found in the literature; i.e., root shaking with isotonic water (NaCl) [39] or with (NaPO_3_)_6_+peptone [40]. The two others were developed in our laboratory and consisted of root shaking (KPBT Sh) or sonication (KPBT So) in KPBT buffer. For each protocol, 2 g of roots coming from the same lettuce were collected and washed 4 times successively. Between each successive wash, roots were rinsed by vortexing in 10 mL of the corresponding buffer (5 mL for the NaCl protocol) and the rinsing solution was added to the previous washing to make sure that all the microorganisms were correctly gathered in the washing Falcon. Then, root washing waters were filtered through sterile cheesecloth to discard root debris. 

### 2.2. Cultivable Microbiota Enumeration

Apple carposphere (collected twice independently from two different commercial apple batches) and lettuce roots washes (collected twice independently at two different washing dates) were serial diluted (1:10 and 1:100) and plated in triplicate by addition of 100 µl of each successive wash suspension per petri dish. Plate media used for apple study case were Reasoner’s 2A agar medium (R2A, yeast extract 0.5 g, proteose peptone 0.5 g, casein hydrolysate 0.5 g, glucose 0.5 g, starch soluble 0.5 g, sodium pyruvate 0.3 g, di-potassium hydrogen phosphate 0.3 g, magnesium sulfate heptahydrate 0.05 g, agar-agar 12 g in 1 L) for bacteria and potato dextrose agar (PDA, 4 g potato peptone, 20 g glucose, and 15 g agar in 1 L) with 100 mg/L of chloramphenicol as specific medium for fungi. Meanwhile, Luria–Bertani agar medium (LB, 10 g tryptone, 5 g yeast extract, 10 g NaCl, 15 g agar in 1 L) was used for lettuces root study case. Colony-forming unit (CFU) enumeration was achieved after Petri dish incubation at 23±2 °C with 16/8 photoperiod over 5–7 days for apple carposphere microbiota and over 3 days for lettuce rhizoplane microbiota. CFU concentrations were calculated by gram of root or by square centimetre of apple fruit. For this, apple fruits surface areas were estimated with a non-linear regression model A = d * x^e^, where x represents the weight (kg) of the four apples, and d, e the combined varieties parameters [41].

### 2.3. Bacterial Microbiota Composition Analysis by 16S rRNA Gene

To gain a better understanding of the effect of the washing method and successive washes on the bacterial composition of the microbiota, the two protocols harvesting the maximum amount of CFU after plating for lettuce (KPBT Sh and KPBT So) and apple (KPBT So and PBS So) were selected for HTS of 16S rRNA gene. Lettuce rhizoplane, or apple carposphere, microbiota harvest was conducted in duplicate, i.e., from two different lettuces, or two different groups of 4 apples (from a single commercial batch). The 4 washes were collected twice independently at 2 different washing dates. For apple carposphere, each successive wash was collected onto 0.2 µm sterile filter (PALL) by vacuum filtration (Bio Rad, Hercules, CA, USA, 900 cu ft/min) under hood, and each filter was processed separately. For root lettuce, the obtained microbiota was concentrated to a pellet by centrifugation at 2350 *g* during 20 min. DNA extractions for each of the four successive washes per protocol were performed by using the FastDNA spin kit (MP Biomedicals, Illkirch-Graffenstaden, France) according to manufacturer’s instructions. The Illumina MiSeq library preparation, sequencing and the quality filtering were performed at DNAVision (Gosselies, Belgium) in two different runs (2 × 300 nt for apple and 2 × 250 nt for lettuce roots). For apple carposphere microbiota, the V3–V4 region of the 16s-rRNA gene was targeted using the modified primers to include Illumina™ adapters: Forward IlumF-Bakt_341F and Reverse IlumR-Bakt_805R [42]. For lettuce roots microbiota, the V1–V3 region was targeted using the Forward 27F and Reverse 534R Illumina primers used in a previous study of aquaponic systems [43].

Reads were demultiplexed and primers were trimmed at the sequencing center and obtained paired-end FASTQ sequences were analyzed with QIIME 2 (q2) version 2019-4 [44]. Quality control and feature table construction was conducted with the q2 DADA2 method without trimming the sequences [45]. Features were classified with the q2 implemented VSEARCH method in the q2 feature-classifier plugin. SILVA_132 at 99% of sequence similarity was used as reference database for the taxonomy. Cytoplasmic contaminations (chloroplast and mitochondria sequences) were discarded with the q2 taxa filter-table script. The q2-diversity core-metrics-phylogenetic plug-in was then used to obtain ecological diversities (alpha and beta) information and for the comparison between the protocols and between the successive four washes. The diversity core-metrics were run on feature table rarefied at 6524 sequences for apple carposphere and 17,463 for lettuce roots. PERMANOVA (999 permutations) Kruskal-Wallis test was used to compare alpha diversity indexes (Observed OTUs, Faith Phylogenetic Diversity (Faith PD), Shannon and Pielou’s Evenness), and beta diversity indexes (Weighted and Unweighted Unifrac distance metrics) were compared by the PERMANOVA (999 permutations) pseudo-F test. Each PERMANOVA *p*-value was automatically corrected in QIIME 2 for multiple analysis of variance. Pseudo count of one were added to the feature tables and the q-2 ANCOM plug-in [46] was used to compare differentially abundant features among washes and between the different protocols. *P*-values with good control of the Benjamini-Hochberg correction (FDR) at 5% type I error rate, are already embedded in the ANCOM test before the final significance based on the empirical distribution of a count random variable called *W* [47].

Additionally, sequences of the four successive washes were pooled for each protocol and normalised at 7,500 sequences for apple fruit and 17,463 for lettuce root. Then, diversity and ANCOM analysis as described above, were used to compare the protocols between themselves and also, the first single wash and the pool of the four washes, for apple fruit and lettuce roots.

Apple carposphere and lettuce root sequences are available on the National Center for Biotechnology Information (NCBI) under the Sequence Read Archive (SRA) accessions PRJNA592976 and PRJNA592958 respectively.

## 3. Results

### 3.1. Apple Carposphere Microbiota

#### 3.1.1. Cultivable Microbiota Enumeration

**Figure 1:**
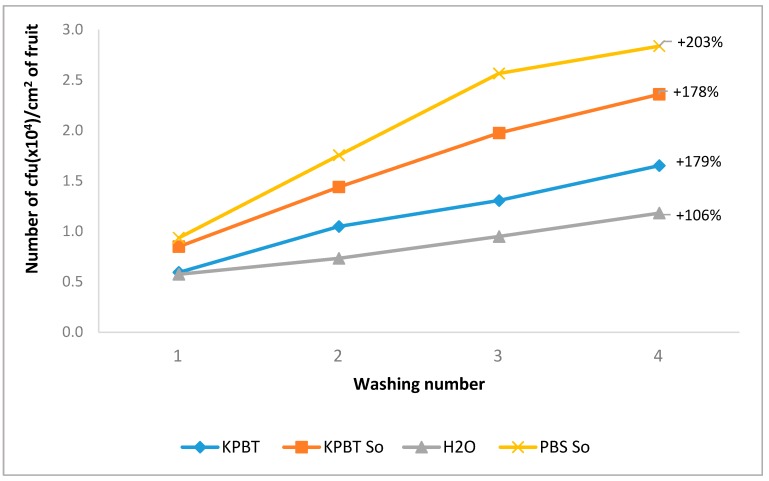
Mean of the cumulative number of CFU per cm^2^ of apple fruit skin for bacteria and fungi counted in the four successive washes for each protocol (KPBT, KPBT So, H_2_O, and PBS So). The percentages on each graph represent the percentage increase of the sum of the four washes compared with the first.

The results of the average cumulative number of the bacterial and fungal CFU/cm^2^ at each successive wash (Figure 1) show that whatever the protocol, the first wash harvested around one-third of the total harvested microbiota (represented by the sum of the four successive washes). After the fourth wash, the increase of percentage of harvested CFU ranged between 106 and 203 % compared to the first wash. In another independent experiment, the apples were successively washed up to eight times with PBS So and the four additional successive washes harvested less than 10% of additional CFU (Figure S1). The PBS So protocol was the most efficient protocol, doubling the performance of water for the first wash.

#### 3.1.2. Bacterial Diversity Analysis by 16S rRNA Gene

The obtained sequences had an average Phred Q30 of 87.7 %, and the full quality control summary is available in interactive view (Figure S2a).

**Figure 2:**
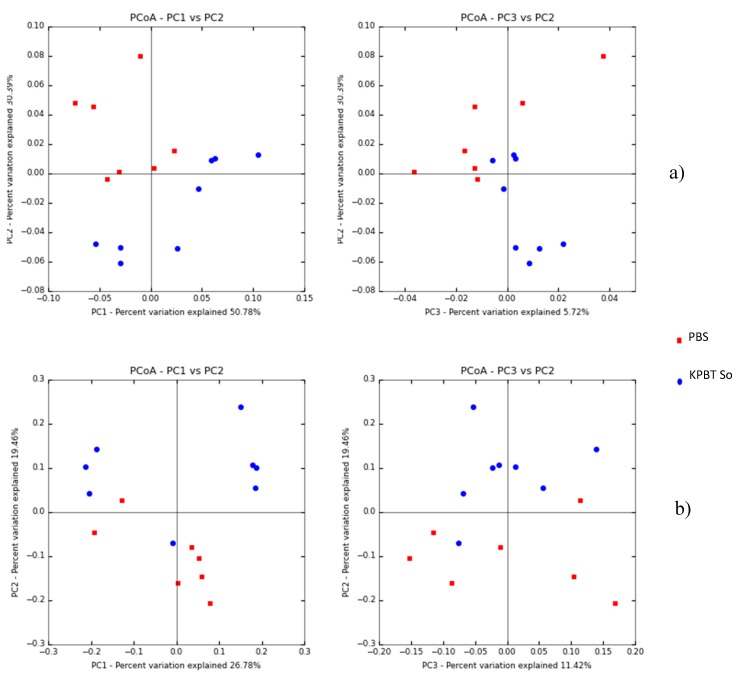
Principal coordinates analysis (PCoA) plots of the weighted (**a**) and unweighted (**b**) Unifrac distance metrics colored by protocols (KPBT So and PBS So) of non-pooled washes. Each dot represents an individual sample from one of the successive washes. All samples were rarefied at 6,524 sequences. Significant differences were observed between each protocol for both weighted and unweighted data.

The comparison among successive washes showed that alpha diversity indexes of Pielou_evenness (*q*-value = 0.005) and Shannon (*q*-value = 0.015) were significantly different between protocols. Also, the beta diversity analysis (both weighted and unweighted) revealed significant differences (*q*-value = 0.011 for both unweighted and weighted Unifrac distance metrics) between the tested protocols (Figure 2). However, whatever the protocol, no difference of alpha or beta diversity was observed between the successive washes.

When washes were pooled (Table 3), the comparison between a single wash and the pool of the four washes showed significant differences of the Faith_Pd (*q*-value = 0.033) and Observed_Otus (*q*-value = 0.032) alpha diversity indexes. Nevertheless, the difference of beta diversity between protocols (Figure 2) disappeared, suggesting a leverage effect when the four washes were pooled, thus allowing non-biased comparison of bacterial community harvested from apple carposphere using different protocols.

**Figure 3:**
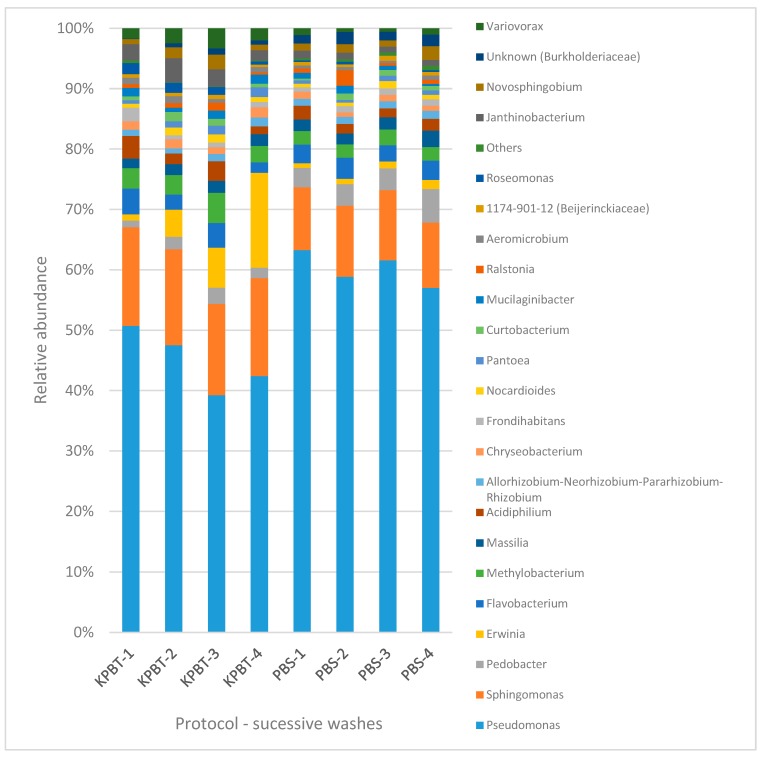
Overview of the taxonomic profile at genera level for the microbiota harvested from apple fruit carposphere. Each column represents all the detected genera of each of the four successive washes with PBS So and KPBT So protocols. Each color represents an OTU. Only the OTUs with high proportion (1% of the total reads) are presented and the rest are grouped as others. The numbers (from 1 to 4) associated to the protocols are successive washes.

Results of the 16s rRNA gene analysis showed that, of the total reads assigned, *Pseudomonas* (50%) and *Sphingomonas* (12%) were the most abundant genera. All the successive washes presented overall a similar taxonomy (Figure 3) per wash whatever the protocol. However, the ANCOM analysis revealed 76 taxa with a significantly different relative abundance between the protocols (Table S3). Moreover, 20 OTUs present with low proportion (e.g., under 0.6% of reads) were not detected in the first wash while they were detected in the further washes at the rarefaction depths mentioned above.

### 3.2. Lettuce Rhizoplane Microbiota

#### 3.2.1. Cultivable Microbiota Enumeration

**Figure 4:**
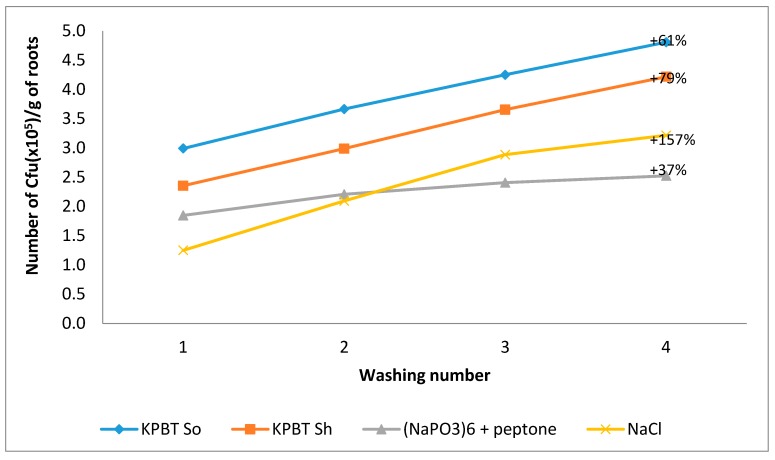
Mean of the cumulative number of CFU per gram of roots for bacteria counted in the four successive washes for each protocol (KPBT So, KPBT Sh, (NaPO_3_)_6_ + Peptone and NaCl). The percentages on each graph represent the percentage increase of the sum of the four washes compared with the first.

The results of the average cumulative number of CFU harvested by each protocol following successive washes are illustrated in Figure 4. As for apples, CFU enumeration showed that the first wash represented the major part of the microbiota recovered. Indeed, the first wash harvested respectively, 64.3%, 53.2%, 64.8%, and 38.2% of the total microbiota (considered as the sum of the four successive washes) for KPBT So, KPBT Si, (NaPO_3_)_6_ + peptone, and NaCl method respectively. Regardless of the number of washes, KPBT So was the most efficient protocol. For example, KPBT So harvested at least twice as much bacteria compared to (NaPO_3_)_6_+peptone. After the fourth wash, the increase in harvested CFU ranged between 37% and 157% compared to the first wash. An increase of 61% was observed with KPBT So.

#### 3.2.2. Bacterial Diversity Analysis by 16S rRNA Gene

The generated sequenced had an average Phred Q30 of 75.5%. Due to an important loss of reads at the merging step of the analysis, only the forward reads were kept for analysis. The full quality control summary is available in interactive view (Figure S2b). After the OTUs table cleaning, 1.9% of reads were unassigned in QIIME 2. Data were rarefied at 17,463 sequences per sample (individual and pooled samples) for the downstream analysis.

**Figure 5:**
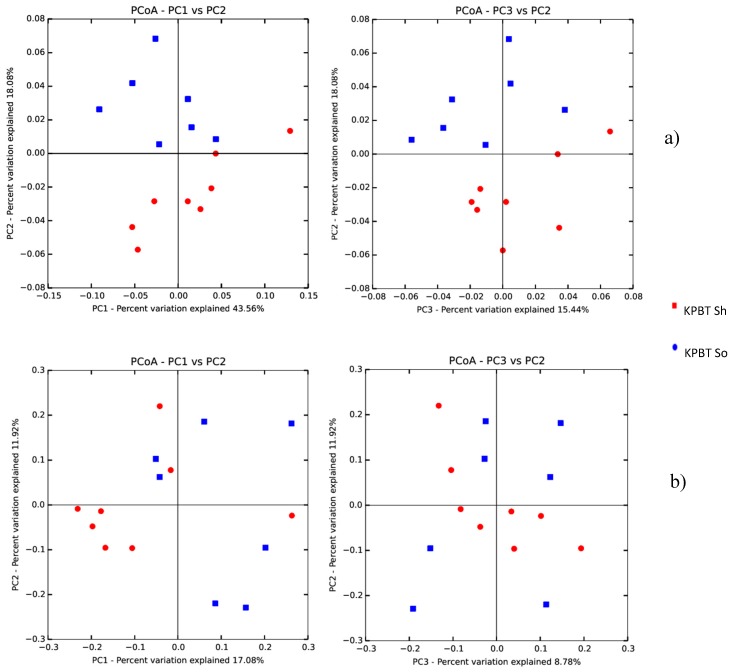
Principal coordinates analysis (PCoA) plots of the weighted (**a**) and unweighted (**b**) Unifrac distance metrics colored by protocols (KPBT So and KPBT Sh) of non-pooled washes. Each dot represents an individual sample from one of the successive washes. All samples were rarefied at 17,463 sequences. Significant differences were observed between each protocol for both weighted and unweighted data.

Significant differences of alpha-diversity with Observed_Otus (*q*-value = 0.028) and faith-pd (*q*-value = 0.037) indexes were observed between the washing methods, i.e., KPBT with shaking or sonication (Table 4). The same difference was highlighted with the β-diversity analysis (*q*-value = 0.025 and 0.018 respectively for unweighted and weighted Unifrac distance metrics) illustrated in Figure 5. However, there was no significant difference of diversity when successive washes or pool of washes were compared between them or with the first wash respectively.

**Figure 6:**
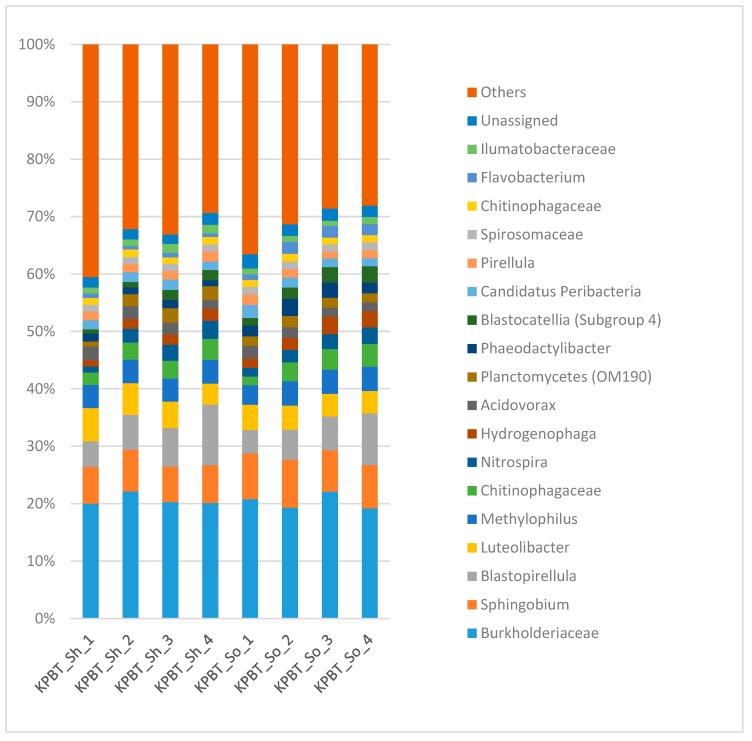
Overview of the taxonomic profile at genera level for the microbiota harvested from lettuce rhizoplane. Each column represents all the detected genera of each of the four successive washes with KPBT So and KPBT Sh protocols. Each color represents an OTU. Only the OTUs with high proportion (1% of the total reads) are presented and the rest are grouped as others. The numbers (from 1 to 4) associated to the protocols are successive washes.

Based on the QIIME 2 taxonomic assignation, the ANCOM analysis did not reveal significant difference of relative abundance between methods, successive washes or between the first wash and four successive washes pooled. This is consistent with the relatively similar composition bar charts displayed on Figure 6. All samples were dominated by the *Burkholderiaceae* family and the *Sphingobium* genus (Figure 6).

## 4. Discussion

In the scientific history, any emerging concept relying on fast moving technologies has been prone to very important bias and errors at its infancy. The origin of bias was initially neglected in microbiome studies but gained more attention recently, including for phytobiome analysis. Over the years, the scientific community developed recommendations and best practices to improve the reliability of the HTS technologies to study plant microbial communities and to promote the comparativeness of the results. The most critical component when analysing the phytobiome is to ensure that the results are representative of the studied microbial community. In this context, phytobiome studies should directly benefit from standardization and recommendations which have been developed for the human microbiome. For instance, specific adaptations have been recommended such as the selection of primers [48], the concentration of DNA [49] or the sequencing technology [16]. Also, bioinformatics ‘best practices’ for microbiome HTS data analyses have been proposed [50] as well as recommendation for data storage and description.

In this paper, a potential bias never explored so far for phytobiome studies was studied: the impact of several successive washes on the harvested microbiota. Currently, there is a diversity of protocols available in the literature, even for a specific plant species and organ (Table 1). The example presented in this paper focuses on two specific cases, but the results warrant further investigation on the efficiency of harvesting strategies. To date, a systematic evaluation of microbiota harvesting efficiency and its potential effect on downstream molecular analyses is missing.

### 4.1. Impact of the Protocol on the Quantity and Diversity of Harvested Microorganisms

Between studies found in the literature, protocols differ in washing buffers (water, phosphate, saline, or phosphate saline), washing time, washing methodology [washing, grinding (therefore also including endophytes), shaking, sonication, or a combination of them]. These variables might greatly impact the outcome of the experiments. For instance, with the grinding of tissues, the endophytes are also collected whilst this is not the case with a simple wash. Thus, it is difficult to compare the results from different publications. For root samples, rhizosphere microbiota is usually harvested by a simple wash to recover the microorganisms loosely attached to the root surface or which were contained in the soil surrounding the roots. While a more aggressive wash of ‘nude’ root (without soil) as met in hydroponics through shaking, glass beads or ultra-sonication bath in a buffer is usually associated with the collection of the rhizoplane microbiota [19,51]. Furthermore, depending on the study, the distinction between rhizosphere and rhizoplane, their definitions or ways to harvest them are sometimes ambiguous [17], especially in the case of plants grown in soilless systems (see Table 1).

The results of the plating of culturable microorganisms showed a huge variability of the number of cells recovered using the tested protocols for both apple and lettuce samples. These results are in accordance with the differences observed in other studies on culturable microorganisms [19,52]. For instance, Rose et al. [53] compared different methods for the recovery of Bacillus anthracis from nonporous surfaces and came with the conclusion that premoistened macrofoam reached the greatest percentage of 43.6% bacteria recovery compared with three other swab materials. Richter-Heitmann et al. [19] found the same trend for rice, clover and bean roots rhizoplane microbiota collection. Their results showed that only 45% of the rhizoplane microorganisms were harvested by vigorous washing, and that additional sonication process increased the detachment up to 78%. In our study, the efficiency of sonication (for lettuce roots) and combined shaking-sonication (for apple fruit) to harvest an increased quantity of microbial cells was also noticed. For apple carposphere, one wash may not be enough if the quantity of culturable microorganisms matters (example of planned glycerol stocks).

Results on culturable microorganisms were confirmed by the 16S rRNA gene HTS analysis. First, the beta diversity analysis in both study cases showed that the harvesting methods significantly influenced the composition of the harvested bacterial community. This may then introduce bias between HTS studies comparison. Secondly, the measured alpha diversity indices (Pielou-evenness and Shannon for apple carposphere and Observed_Otus and Faith_pd for lettuces’ roots) also confirmed a significant difference in the diversity of the harvested bacterial communities between washing protocols. With regard to Richter-Heitmann et al. [19], they did not observe differences with community fingerprinting of 16S rRNA gene by T-RFLP between washing methods on rice roots. However, they interpret these findings by suggesting that root morphological parameters strongly influence the efficiency of the washing method as differences were observed with other plant roots (bean and clover).

### 4.2. Impact of Successive Washes on the Quantity and Diversity of Harvested Microorganisms

The compositions of the successive washes were compared thanks to HTS tools. No qualitative differences were found amongst the successive washes either for apple carposphere or lettuce root for the same protocol. Indeed, the ANCOM test did not highlight any OTU which could be present in different abundances between the successive washes. This result indicates that for the same protocol, single washes can be fairly compared by 16S rRNA gene HTS analysis. However, concerning the quantity of microorganisms collected, the first wash of apple carposphere or lettuce roots microbiota harvested less than half of the microorganisms collected after four successive washes, whatever the protocol. For apple carposphere [32] and lettuce roots [35], the taxa detected in this study were generally consistent with literature until the class level but started to differ at a more precise taxonomic level. However, even with similar hypervariable regions (V1–V3 for lettuce roots and V3–V4 for apple fruit) and resembling phosphate buffers (for apple fruit), the alpha-diversity indices obtained in this study were much higher than the ones found in the literature for both apple carposphere [32] and lettuce roots [35].

This study has shown that pooling the successive washes allowed to increase the amount of culturable microorganisms with variable yields. Interestingly, the alpha- and beta-diversity between protocols were not significantly different anymore when the sequences of the four successive washes were pooled together for apple carposphere or on lettuce roots. In additin, no taxon was significantly different between the tested protocols for apple. Therefore, pooling several outwashes appears to allow fair comparison between studies using different protocols. Moreover, the richness indices (Faith_PD and Observed_Otus) of the pooled washes were significantly higher than the single wash (Table 3b) for apple epiphytic microbiota (after normalization at the same sequencing depth). This explains why 20 OTUs with low proportion were not detected in the first washes while they were detected with further washes. We thus suggest that the pooling of successive washes could be a good solution to reach a improved resolution of the bacterial community colonizing a biological replicate, and that it might potentially reduce high variability between biological replicates and studies, especially for HTS studies where there are no or few repetitions. It could also leverage the detection of taxa strongly attached to the plant material.

In addition, through the concentration effect of the quantity of microorganisms harvested with the successive washes pooled, it has the potential to limit bias linked to microbial DNA contaminations present in the extraction kits [54]. Though only bacterial population were targeted in the 16S rRNA gene analysis, similar results need to be confirmed for fungal populations. Thus, there is also a need to pay attention to these parameters in further studies when harvesting microbial cells from plants.

## 5. Conclusions

These two study cases indicate that the washing protocol significantly influences the quantity and the bacterial diversity of microorganisms harvested and that four successive washes can increase up to three-times the quantity of harvested microorganisms. In the tested conditions, the washing protocol significantly influenced bacterial beta diversity for apple carposphere and lettuce rhizoplane. There were no significant differences in bacterial alpha and beta diversity and OTU abundances from apple carposphere and lettuce roots between washing protocols after pooling the four successive washes together, potentially indicating that each protocol repeated four times harvested nearly completely the epiphytic microbiota. The richness of the bacterial population (for Faith_PD and Observed_Otus indexes) was also significantly higher with four washes comparing to a single wash, but only in the case of apple carposphere. Based on these results and the literature, we therefore recommend to carefully evaluate the opportunity to wash each sample several times in order to harvest plant epiphytic microbiota with limited bias. Such evaluation is an important, although currently neglected step toward a better comparability between phytobiome studies.

## Figures and Tables

**Table 1:** Diversity of protocol for harvesting microbiomes from apple carposphere and lettuce roots.

Host	Buffer	Technic Use	Purpose	Reference
Apple	Malt extract + glucose + antibiotic	Culture of fruit slice shaken at 100 rpm (3–10 days)	Plating	[20]
	Sterile deionized water	Two washes (by dipping)	Biological assay	[21]
	Phosphate buffer, pH 6.5	Use of second wash; 30 s sonication + 100 rpm shaking for 10 min (the first washing with no sonication is thrown)	Plating	[22]
	Phosphate buffer, pH 6.5	Use of second wash; 10 min sonication + 150rpm shaking for 10 min (the first wash with no sonication is thrown)	Plating	[23]
	Sterile water	100 rpm shaking for 10 min	Plating	[24]
	Double deionized water (DDI)	2 min mixing the sample + 1 min sonication on each side	454-amplicon sequencing	[25]
	Deionized sterile water	5 min sonication	Amplicon sequencing	[26]
	Phosphate buffer, pH = 7	Fruit dissection + shaking for 20 min at 150 rpm + 10 min sonication	Plating	[27]
	Phosphate buffer, pH = 6.5	Rinsing by shaking at 120 rpm for 20 min	Plating	[28]
	Water and phosphate buffer, pH 6.5	Rinsing first time in water; and once with sonication bath with phosphate buffer	Plating	[29]
	No buffer	Wiping the fruit surface with moistened cotton swab	Amplicon sequencing	[30,31]
	Phosphate buffer, pH 6.8	Rinsing by shaking at 120 rpm for 2 h	Amplicon sequencing	[32]
Lettuce in soil	Sterile saline water	Shaking by rotary shaker at 307 rpm with glass bead for 1 h	PCR-DGGE,	[33]
	Sterile 0.9% NaCl + 0.02% Silwet L-77 solution	Vortexing 2 times for 15s	Plating	[34]
Lettuce in aquaponics	Sterile ultrapure water	Vortexing for 2 min followed by 5 min ultrasonic bath	Illumina sequencing	[35]
	Sterile peptone phosphate buffer (1g peptone + 1.21 g K_2_HPO_4_ + 0.34 g KH_2_PO_4_ + 1 L deionized water	Crushing with a tissue lyser	T-RFLP	[36]

**Table 2:** Methods description of apple carposphere harvesting (A) and lettuce rhizoplane harvesting (B).

Treatment Label	Material	Solution	Treatment 1	Duration 1	Intensity 1	Treatment 2	Duration 2	Intensity 2	Reference
KPBT	4 apples	1 L of a sterile 0.05M potassium phosphate buffer + 0.1% tween80 pH 6.5	Shaking	20 min	120 rpm	-	-	-	[28]
KPBT So	4 apples	1 L of a sterile 0.05 M potassium phosphate buffer + 0.1% tween80 pH 6.5	Sonication in ultra-bath	10 min	-	Shaking	20 min	120 rpm	Modified from [28]
PBS So	4 apples	1 L of sterile Phosphate Buffer Saline, pH 7.4	Sonication in ultra-bath	20 min	-	Shaking	20 min	120 rpm	Modified from [23]
H_2_O	4 apples	1 L of sterile distilled water	Shaking	10 min	120 rpm	-	-	-	[24,37]
**A**									
**Treatment Label**	**Material**	**Solution**	**Treatment 1**	**Duration 1**	**Intensity 1**	**Reference**			
(NaPO3)6 + peptone	2 g of roots	30 mL of a 2 g/L (NaPO3)6 + 1 g/L peptone sterile solution	Shaking	20 min	200 rpm	[40]			
NaCl	2 g of roots	5 mL of isotonic sterile water (0.85% NaCl)	Shaking	10 min	150 rpm	[39]			
KPBT Sh	2 g of roots	30 mL of a sterile 0.05 M potassium phosphate buffer + 0.05% tween80 pH 6.5	Shaking	20 min	150 rpm	Intern protocol			
KPBT So	2 g of roots	30 mL of a sterile 0.05 M potassium phosphate buffer + 0.05% tween80 pH 6.5	Ultra-bath sonication	10 min	-	Intern protocol			
**B**									

**Table 3:** Alpha-diversity comparisons (pairwise Kruskal–Wallis, *q*-value) between the protocols, between successive washes before pooling of washes and after the pooling of washes of apple fruit carposphere; ns = not statistically significant; pd = phylogenetic diversity; So = sonication. All the successive washes were pairwise compared.

	Faith_pd	Pielou_Eveness	Observed_Otus	Shannon
KPBT So	5.13	0.85	184	6.36
PBS So	5.14	0.78	172	5.76
Comparison between KPBT So and PBS So	ns	0.005	Ns	0.015
First wash	5.37	0.80	193	6.06
Second wash	5.15	0.82	180	6.13
Third wash	5.13	0.82	175	6.11
Fourth wash	4.89	0.81	165	5.92
Pairwise comparisons (six) between the successive washes	ns for all	ns for all	ns for all	ns for all
(a) Comparison of non-pooled washes (rarefied at 6524 sequences);				
	**Faith_pd**	**Pielou_Eveness**	**Observed_Otus**	**Shannon**
KPBT So	6.29	0.83	251	6.59
PBS So	5.92	0.75	229	5.82
Comparison between KPBT So and PBS So	ns	ns	ns	ns
First wash	5.24	0.78	189	5.91
Pooled of the four washes	6.71	0.78	276	6.33
Comparisons between the first wash and the pooled of the four successive washes	0.033	ns	0.032	ns
(b) Comparison of pooled washes (rarefied at 7500 sequences).				

**Table 4:** Alpha diversity comparison (pairwise Kruskal-Wallis *q*-value) between the protocols, between successive washes before pooling of washes and after the pooling of washes of lettuce rhizoplane; ns = not statistically significant; pd= phylogenetic diversity; Sh = shaking; So = sonication.

	Faith_PD	Pielou_Eveness	Observed_Otus	Shannon
KPBT Sh	106	0.73	764	6.99
KPBT So	85	0.73	622	6.75
Pairwise comparison between KPBT Sh and KPBT So	0.037	ns	0.028	ns
First wash	100	0.76	750	7.19
Second wash	98	0.73	780	6.96
Third wash	88	0.73	604	6.77
Fourth wash	100	0.71	670	6.65
Pairwise comparisons (six) between the successive washes	ns for all	ns for all	ns for all	ns for all
(a) Comparison of non-pooled washes (rarefied at 17,463 sequences); all the successive washes were pairwise compared				
	**Faith_PD**	**Pielou_Eveness**	**Observed_Otus**	**Shannon**
KPBT Sh	120	0.73	925	7.2
KPBT So	80	0.72	609	6.95
Pairwise comparison between KPBT Sh and KPBT So	ns	ns	ns	ns
First wash	98	0.76	755	7.2
Pooled of the four washes	116	0.72	885	7.00
Comparisons between the first wash and the pooled of the four successive washes	ns	ns	ns	ns
(**b**) Comparison of pooled washes (rarefied at 17,463 sequences)				

## Supplementary Materials

Supplementary materials can be found at https://www.mdpi.com/2076-2607/8/3/342/s1.
