# Supplementary material for: Standardization of Plant Microbiome Studies: Which Proportion of the Microbiota is Really Harvested?
Source: Microorganisms. 2020 Feb 28;8(3):342. doi: 10.3390/microorganisms8030342 (PMC7142977; doi:10.3390/microorganisms8030342)
Supplement: Supplementary file 1 [file microorganisms-08-00342-s001.zip › Supplementary material_1_Evolution of the relative abundance of the cumulated number of CFU upt ot 8 washes for apple.docx]

Supplementary materials 1

Evolution of the relative abundance of the cumulated number of CFU of bacteria and fungi harvested up to eight successive washes for the PBS-So protocol. The cumulated number of CFU after the eight washes (T) was considered as the total number of CFU that can be harvested. The relative abundance of CFU for each wash (W) was calculated by dividing W over T.
